# Pre-referral GP consultations in patients subsequently diagnosed with rarer cancers: a study of patient-reported data

**DOI:** 10.3399/bjgp16X683977

**Published:** 2016-02-26

**Authors:** Silvia C Mendonca, Gary A Abel, Georgios Lyratzopoulos

**Affiliations:** Cambridge Centre for Health Services Research, Institute of Public Health, University of Cambridge, Cambridge.; Cambridge Centre for Health Services Research, Institute of Public Health, University of Cambridge, Cambridge.; Cancer Research UK Health Behaviour Research Centre, Department of Epidemiology and Public Health, University College London, London.

**Keywords:** consultation, diagnosis, investigation, neoplasm, primary care, referral

## Abstract

**Background:**

Some patients with cancer experience multiple pre-diagnostic consultations in primary care, leading to longer time intervals to specialist investigations and diagnosis. Patients with rarer cancers are thought to be at higher risk of such events, but concrete evidence of this is lacking.

**Aim:**

To examine the frequency and predictors of repeat consultations with GPs in patients with rarer cancers.

**Design and setting:**

Patient-reported data on pre-referral consultations from three English national surveys of patients with cancer (2010, 2013, and 2014), pooled to maximise the sample size of rarer cancers.

**Method:**

The authors examined the frequency and crude and adjusted odds ratios for ≥3 (versus 1–2) pre-referral consultations by age, sex, ethnicity, level of deprivation, and cancer diagnosis (38 diagnosis groups, including 12 rarer cancers without prior relevant evidence).

**Results:**

Among 7838 patients with 12 rarer cancers, crude proportions of patients with ≥3 pre-referral consultations ranged from >30.0% to 60.0% for patients with small intestine, bone sarcoma, liver, gallbladder, cancer of unknown primary, soft-tissue sarcoma, and ureteric cancer. The range was 15.0–30.0% for patients with oropharyngeal, anal, parotid, penile, and oral cancer. The overall proportion of responders with any cancer who had ≥3 consultations was 23.4%. Multivariable logistic regression indicated concordant patterns, with strong evidence for variation between rarer cancers (*P* <0.001).

**Conclusion:**

Patients with rarer cancers experience pre-referral consultations at frequencies suggestive of middle-to-high diagnostic difficulty. The findings can guide the development of new diagnostic interventions and ‘safety-netting’ approaches for symptomatic presentations encountered in patients with rarer cancers.

## INTRODUCTION

Most patients with cancer first present with symptoms, typically to non-specialists;^1^,^2^ consequently, ongoing initiatives in several countries aim to improve diagnostic timeliness for these patients.^3^–^6^ Promptly suspecting the diagnosis in patients with rarer cancers who are symptomatic may be particularly challenging, given their overall rarity and heterogeneous nature. In recent years, international initiatives have focused attention on the management of rarer cancers but improvements in diagnosis are also needed.^7^

A key marker of diagnostic timeliness in patients who are symptomatic and subsequently diagnosed with cancer is the number of consultations they have with a GP before a specialist referral is made, which is highly correlated with the ‘primary care interval’ (time from first presentation to referral).^8^,^9^ National audit data indicate primary care intervals of approximately 1, 1.5, and 3 months in patients who experience 3, 4, and ≥5 or more pre-referral consultations.

Describing variation in pre-referral consultations between different patient groups and cancers can provide insights into aetiological mechanisms responsible for diagnostic delay, and help inform future policies and research.^10^,^11^ In some cancers, most patients present with symptoms of relatively high predictive value; for example, in the case of breast cancer this is a lump, or in melanoma a visible skin lesion.^10^ In some other cancers, most patients present with poorly predictive symptoms; as an example, most patients with pancreatic cancer and multiple myeloma present with abdominal or musculoskeletal pain, respectively.^12^,^13^ Consequently, variation in the proportion of patients who experience multiple consultations reflects differences in the ‘symptom signature’ of different cancers.^10^,^11^ Based on these considerations, it has been suggested that different cancers can be broadly categorised for their diagnostic difficulty, based on the respective frequency of multiple (≥3) consultations: cancers in which ≥30% of patients experience multiple consultations are considered ‘harder to suspect’, whereas those for which <15% of patients have multiple consultations are classed as ‘easier to suspect’.^10^

As cancer is a heterogeneous disease, evidence about the burden of pre-diagnostic consultations across the range of common and rare cancers is desirable. Nonetheless, currently available evidence (chiefly based on information from responders to a national patient survey in England) excludes patients with several rarer cancers.^11^ Pooling of data from different waves of surveys of patients with cancer can help to overcome sample size limitations for patients with rarer cancers. As a result, a study was conducted with the principal objective of addressing current evidential gaps about the burden of multiple pre-referral consultations in patients with rarer cancers.

**How this fits in**

Patients subsequently diagnosed with rarer cancers are often thought to experience multiple pre-referral primary care consultations, but evidence for this assertion is limited. The frequency and predictors of multiple pre-diagnostic consultations in over 7800 patients with 12 rarer cancers were examined. For patients with small intestine, bone sarcoma, liver, gallbladder, cancer of unknown primary, soft-tissue sarcoma, and ureteric cancer, crude proportions of patients with ≥3 pre-diagnostic consultations of >30.0–60.0% were observed. The findings support the development of decision support tools, new diagnostic pathways, and ‘safety-netting’ approaches for patients with possible symptoms of rarer cancers.

## METHOD

Data from three waves of the *National Cancer Patient Experience Survey* (2010, 2013, and 2014), which surveyed patients treated for cancer in English NHS hospitals during sampling periods of 3 months, were analysed.^14^–^16^ All three surveys were commissioned by the UK Department of Health and carried out by Quality Health, a specialist survey provider, using identical sampling methods. Survey questions were cognitively validated on samples of volunteer patients.

Patients were sent a survey questionnaire by post a few weeks after discharge and after vital status checks, with up to two reminders sent to non-responders. Response rates were 67%, 64%, and 64% respectively for the 2010, 2013, and 2014 surveys. Anonymous data from the surveys, as used in this study, are made available for research purposes from the UK Data Archive.

There is no universal definition of what constitutes a rarer cancer.^7^ In this article, the authors specifically focus on rarer cancers without prior published evidence, 10 of which have an annual incidence of <4000/year in England or <1.5% of the annual incidence of all malignant neoplasms, excluding non-melanoma skin cancer. Data from patients with any cancer were included to support direct comparisons between these patient groups and to better contextualise the findings.

### Data analysis

#### Outcome and exposure variables

The information used was provided in response to the first question in the questionnaire: ‘Before you were told you needed to go to hospital about cancer, how many times did you see your GP (family doctor) about the health problem caused by cancer?’ Possible answers were:

- none — I did not see my GP before going to hospital;
- once;
- twice;
- three or four times;
- ≥5 times; and
- don’t know/can’t say.

For the analysis, the binary outcome of ≥3 versus 1 or 2 pre-referral consultations were used; patients who responded that they had not seen their GP before going to hospital or responded with ‘Don’t know/can’t say’ were excluded. This binary categorisation is concordant with how data from this survey are reported publicly, and reflects the consideration that some second appointments are generated by the need to review the findings of investigations ordered at the first consultation.^10^,^14^–^17^ Exposure variables considered were:

- age;
- sex;
- deprivation group (an ecological measure of socioeconomic status based on quintile groups of the Index of Multiple Deprivation scores of the lower super output area of patients’ residence^18^);
- *International Classification of Diseases*, 10^th^ edition diagnosis code based on hospital records (Table 1);
- self-assigned Office for National Statistics classification of ethnic group (based on responses to a survey item^19^); and
- survey wave.

**Table 1.** ICD-10 definitions of cancer types used in analysis ^a^

**Cancer type**	**ICD-10 code**	**ICD-10 code description**	**Incident count (2012), England**
***Oropharyngeal***	*C01, C09, C10*	*Malignant neoplasms of base of tongue (C01), tonsil (C09), and oropharynx (C10)*	*2037*
***Oral***	*C02, C03, C04, C06*	*Malignant neoplasm of other and unspecified parts of tongue (C02), gum (C03), floor of mouth (C04), and palate (C06)*	*2544*
***Parotid***	*C07, C08*	*Malignant neoplasm of parotid gland (C07) and other and unspecified major salivary glands (C08)*	*556*
Oesophageal	C15	Malignant neoplasm of oesophagus	7243
Stomach	C16	Malignant neoplasm of stomach	5637
***Small intestine***	*C17*	*Malignant neoplasm of small intestine*	*1065*
Colon	C18	Malignant neoplasm of colon	22 401
Rectal	C19, C20	Malignant neoplasm of recto-sigmoid junction (C19) and of rectum (C20)	11 921
***Anal***	*C21*	*Malignant neoplasm of anus and anal canal*	*1043*
***Liver***	*C22*	*Malignant neoplasm of liver and intrahepatic bile ducts*	*3867*
***Gallbladder***	*C23*	*Malignant neoplasm of gallbladder*	*686*
Pancreatic	C25	Malignant neoplasm of pancreas	7371
Laryngeal	C32	Malignant neoplasm of larynx	1876
Lung	C33, C34	Malignant neoplasm of trachea (C33), and bronchus and lung (C34)	35 903
***Bone sarcoma***	*C40, C41*	*Malignant neoplasm of bone and articular cartilage of limbs (C40) and other and unspecified sites (C41)*	*412*
Melanoma	C43	Malignant melanoma of skin	11 281
Mesothelioma	C45	Mesothelioma	2347
***Soft-tissue sarcoma***	*C49*	*Malignant neoplasm of other connective and soft tissue*	*1521*
Breast	C50	Malignant neoplasm of breast	42 773
Vulval/vaginal	C51, C52	Malignant neoplasm of vulva (C51) and vagina (C52)	1262
Cervical	C53	Malignant neoplasm of cervix uteri	1262
Endometrial	C54, C55	Malignant neoplasm of corpus uteri (C54), malignant neoplasm of uterus, unspecified (C55)	7192
Ovarian	C56	Malignant neoplasm of ovary	5582
***Penile***	*C60*	*Malignant neoplasm of penis*	*505*
Prostate	C61	Malignant neoplasm of prostate	37 136
Testicular	C62	Malignant neoplasm of testis	1874
Renal	C64	Malignant neoplasm of kidney, except renal pelvis	7366
***Ureteric***	C65, C66	Malignant neoplasm of renal pelvis (C65) and ureter (C66)	1082
Bladder	C67	Malignant neoplasm of bladder	9124
Brain	C71	Malignant neoplasm of brain	3959
Thyroid	C73	Malignant neoplasm of thyroid gland	2595
***Cancer of unknown primary***	*C77, C78, C79, C80*	*Secondary and unspecified malignant neoplasm of lymph nodes (C77), secondary malignant neoplasm of respiratory and digestive organs (C78), secondary malignant neoplasm of other and unspecified sites (C79), and malignant neoplasm, without specification of site (C80)*	*7965*
Hodgkin lymphoma	C81	Hodgkin lymphoma	1555
Non-Hodgkin lymphoma	C82, C83, C85	Follicular (nodular) non-Hodgkin lymphoma (C82), diffuse non-Hodgkin lymphoma (C83), other and unspecified types of non-Hodgkin lymphoma (C85)	10 144
Multiple myeloma	C90	Multiple myeloma and malignant plasma cell neoplasms	4190
Leukaemia	C91, C92, C93, C94, C95	Lymphoid leukaemia (C91), myeloid leukaemia (C92), monocytic leukaemia (C93), other leukaemias of specified cell type (C94), other leukaemias of unspecified cell type (C95)	7354
Ductal carcinoma *in situ*	D05	Carcinoma *in situ* of breast	5517
Other cancers	All other codes		

a The number of incident cases in England is also provided as a measure of cancer frequency; bold italics denote rarer cancers without prior relevant evidence.

*ICD* =*10* = International Classification of Diseases*, 10^th^ edition*.

#### Analysis sample derivation

Of 206 591 responders to all three surveys, the analysis sample was restricted a priori to patients who indicated (in response to a survey question) that they were diagnosed with cancer during the year before completing the survey (62.4% of the initial responders’ sample). This was done to minimise the potential of ‘double-counting’ the few responders who may have been treated during the sampling periods of more than one survey.

**Figure 1.**
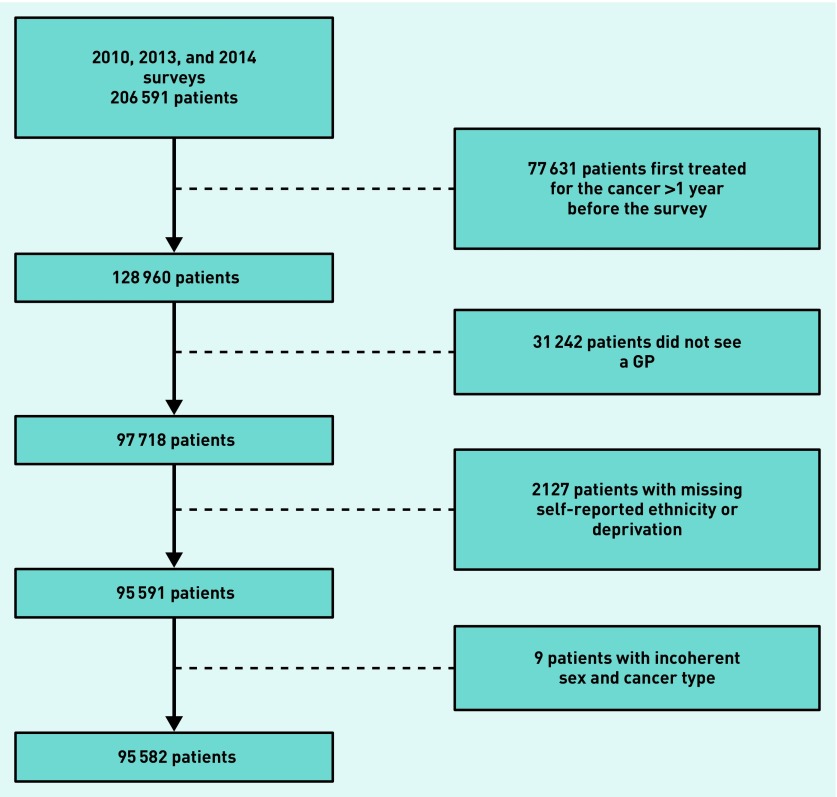
***Analysis sample derivation flowchart.***

Among patients who saw the GP at least once, there was complete information on age, sex, and cancer diagnosis, but 2.2% had missing information on ethnicity or deprivation group — these records were excluded from subsequent analyses, resulting in an analysis sample of 95 582 patients. Figure 1 outlines the derivation of the analysis sample.

#### Statistical analysis

The crude proportion of patients who had ≥3 consultations are described by exposure variable category; that is, by age group, sex, ethnicity, deprivation group, and cancer diagnosis. Subsequently, using logistic regression, the crude and adjusted (for all above variables) odds ratios (ORs) for ≥3 pre-referral consultations are reported. Standard errors were calculated with a robust estimator. Rectal cancer was used as the reference category for cancer, as a result of it having a large sample size and occurring in both sexes. Additionally, crude proportions of patients for each individual category of number of pre-referral consultations by cancer diagnosis are described. All analyses were performed using Stata (version 13).

## RESULTS

After exclusions, 95 582 responders were included in the analysis, of whom 7838 were patients with the following 12 rarer cancers: oropharyngeal, oral, parotid, small intestine, anal, liver, gallbladder, bone sarcoma, soft-tissue sarcoma, penile, ureteric, and cancer of unknown primary. Overall, 22 387 (23.4%) patients had ≥3 consultations.

**Figure 2.**
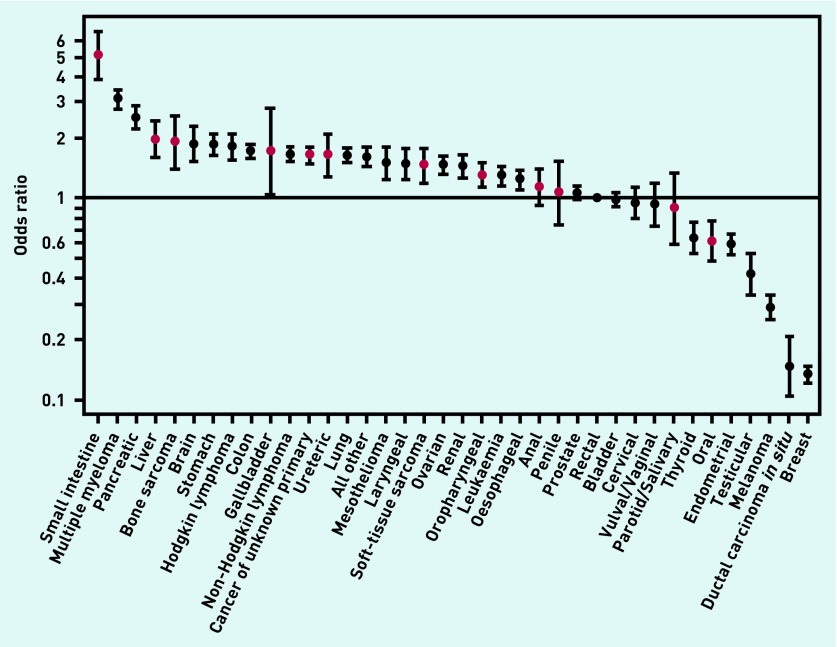
***Odds ratios for ≥3 GP consultations before hospital referral, by cancer type. Central estimates for 12 rarer cancers without prior relevant published evidence shown in red.***

There was strong evidence for very large variation in crude proportions, and crude and adjusted ORs of ≥3 pre-referral consultations between patients with different cancers (*P* <0.001, Table 2, Figure 2). The proportion of patients with ≥3 consultations was highest for small intestine (60.1%) and multiple myeloma (47.2%), and lowest for melanoma (8.3%), ductal carcinoma *in situ* (5.5%), and breast cancer (5.0%). There was a notably large — 40-fold — difference in the adjusted odds between small intestine cancer and breast cancer in terms of patients having ≥3 consultations.

**Table 2.** Crude proportions, crude ORs, and adjusted ORs for ≥3 pre-diagnostic GP consultations, by cancer^a^

**Patient characteristics**	**All responders who saw GP at least once, *n***	**Responders with ≥3 consultations, *n* (%)**	**Crude OR (95% CI)**	***P*-value**	**Adjusted OR (95% CI)**	***P*-value**
**Cancer diagnosis**						
***Small intestine***	*218*	*131 (60.1)*	*5.18 (3.92 to 6.84)*		*5.09 (3.85 to 6.73)*	
Multiple myeloma	2052	969 (47.2)	3.08 (2.77 to 3.42)		3.08 (2.77 to 3.43)	
Pancreatic	1137	485 (42.7)	2.56 (2.24 to 2.92)		2.52 (2.21 to 2.89)	
***Liver***	*451*	*167 (37.0)*	*2.02 (1.65 to 2.47)*		*1.96 (1.60 to 2.40)*	
***Bone sarcoma***	*196*	*81 (41.3)*	*2.42 (1.81 to 3.24)*		*1.89 (1.39 to 2.56)*	
Brain	474	186 (39.2)	2.22 (1.83 to 2.70)		1.86 (1.53 to 2.26)	
Stomach	1828	623 (34.1)	1.78 (1.58 to 2.00)		1.86 (1.65 to 2.09)	
Hodgkin lymphoma	969	417 (43.0)	2.60 (2.25 to 2.99)		1.79 (1.54 to 2.08)	
Colon	8053	2640 (32.8)	1.68 (1.55 to 1.81)		1.71 (1.58 to 1.85)	
***Gallbladder***	*66*	*23 (34.8)*	*1.84 (1.10 to 3.06)*		*1.70 (1.03 to 2.80)*	
Non-Hodgkin lymphoma	5523	1844 (33.4)	1.72 (1.59 to 1.87)		1.65 (1.52 to 1.80)	
***Cancer of unknown primary***	*3537*	*1189 (33.6)*	*1.74 (1.59 to 1.91)*		*1.63 (1.49 to 1.80)*	
***Ureteric***	*331*	*100 (30.2)*	*1.49 (1.17 to 1.90)*		*1.63 (1.28 to 2.08)*	
Lung	6405	2068 (32.3)	1.64 (1.51 to 1.78)		1.63 (1.50 to 1.77)	
All other	2281	753 (33.0)	1.70 (1.52 to 1.89)		1.60 (1.43 to 1.78)	
Mesothelioma	641	180 (28.1)	1.34 (1.12 to 1.61)		1.49 (1.24 to 1.80)	
Laryngeal	667	198 (29.7)	1.45 (1.22 to 1.73)		1.49 (1.24 to 1.78)	
***Soft-tissue sarcoma***	*508*	*162 (31.9)*	*1.61 (1.32 to 1.96)*		*1.46 (1.19 to 1.78)*	
Ovarian	2717	913 (33.6)	1.74 (1.57 to 1.93)	<0.0001	1.45 (1.31 to 1.61)	<0.0001
Renal	1436	431 (30.0)	1.48 (1.30 to 1.68)		1.43 (1.26 to 1.63)	
***Oropharyngeal***	*1155*	*338 (29.3)*	*1.42 (1.24 to 1.64)*		*1.30 (1.13 to 1.50)*	
Leukaemia	2043	582 (28.5)	1.37 (1.22 to 1.54)		1.29 (1.14 to 1.45)	
Oesophageal	2887	737 (25.5)	1.18 (1.06 to 1.31)		1.23 (1.11 to 1.37)	
***Anal***	*495*	*133 (26.9)*	*1.26 (1.03 to 1.56)*		*1.14 (0.92 to 1.40)*	
***Penile***	*174*	*38 (21.8)*	*0.96 (0.67 to 1.38)*		*1.07 (0.74 to 1.53)*	
Prostate	8538	1863 (21.8)	0.96 (0.89 to 1.04)		1.06 (0.97 to 1.15)	
Rectal	5616	1265 (22.5)	Ref		Ref	
Bladder	6837	1397 (20.4)	0.88 (0.81 to 0.96)		0.99 (0.90 to 1.07)	
Cervical	706	203 (28.8)	1.39 (1.17 to 1.65)		0.95 (0.79 to 1.14)	
Vulval/vaginal	405	95 (23.5)	1.05 (0.83 to 1.34)		0.93 (0.73 to 1.18)	
***Parotid***	*140*	*32 (22.9)*	*1.02 (0.68 to 1.52)*		*0.89 (0.60 to 1.34)*	
Thyroid	940	197 (21.0)	0.91 (0.77 to 1.08)		0.63 (0.53 to 0.76)	
***Oral***	*567*	*94 (16.6)*	*0.68 (0.54 to 0.86)*		*0.61 (0.49 to 0.77)*	
Endometrial	3255	549 (16.9)	0.70 (0.62 to 0.78)		0.59 (0.53 to 0.66)	
Testicular	665	96 (14.4)	0.58 (0.46 to 0.73)		0.42 (0.33 to 0.53)	
Melanoma	3470	289 (8.3)	0.31 (0.27 to 0.36)		0.29 (0.25 to 0.33)	
Ductal carcinoma *in situ*	650	36 (5.5)	0.20 (0.14 to 0.28)		0.15 (0.10 to 0.21)	
Breast	17 549	883 (5.0)	0.18 (0.17 to 0.20)		0.13 (0.12 to 0.15)	

**Sex**						
Male	45 151	11 613 (25.7)	Ref	<0.0001	Ref	<0.0001
Female	50 431	10 774 (21.4)	0.78 (0.76 to 0.81)		1.24 (1.19 to 1.29)	

**Age group, years**						
16–24	536	231 (43.1)	2.40 (2.02 to 2.85)		2.12 (1.75 to 2.56)	
25–34	1754	508 (29.0)	1.29 (1.16 to 1.44)		1.82 (1.61 to 2.06)	
35–44	5277	1046 (19.8)	0.78 (0.73 to 0.84)		1.46 (1.34 to 1.58)	
45–54	13 113	2937 (22.4)	0.91 (0.87 to 0.96)		1.38 (1.31 to 1.46)	
55–64	22 789	5881 (25.8)	1.10 (1.06 to 1.15)		1.18 (1.14 to 1.23)	
65–74	30 517	7327 (24.0)	Ref	<0.0001	Ref	<0.0001
75–84	18 442	3860 (20.9)	0.84 (0.80 to 0.88)		0.84 (0.81 to 0.88)	
≥85	3154	597 (18.9)	0.74 (0.67 to 0.81)		0.78 (0.71 to 0.86)	

**Ethnic group**						
White	91 824	21 024 (22.9)	Ref		Ref	
Mixed	456	161 (35.3)	1.84 (1.52 to 2.23)		1.79 (1.44 to 2.23)	
Asian^b^	1662	611 (36.8)	1.96 (1.77 to 2.17)	<0.0001	2.20 (1.96 to 2.46)	<0.0001
Black	1280	483 (37.7)	2.04 (1.82 to 2.29)		2.17 (1.91 to 2.47)	
Chinese	236	71 (30.1)	1.45 (1.10 to 1.91)		1.30 (0.96 to 1.76)	
Other	124	37 (29.8)	1.43 (0.97 to 2.10)		1.57 (1.02 to 2.43)	

**Deprivation group^c^**						
Affluent	22 351	4883 (21.8)	Ref		Ref	
Deprivation group 2	22 644	5127 (22.6)	1.05 (1.00 to 1.09)		1.04 (0.99 to 1.09)	
Deprivation group 3	20 446	4743 (23.2)	1.08 (1.03 to 1.13)	<0.0001	1.05 (1.00 to 1.10)	<0.0001
Deprivation group 4	16 868	4117 (24.4)	1.16 (1.10 to 1.21)		1.08 (1.03 to 1.14)	
Deprived	13 273	3517 (26.5)	1.29 (1.23 to 1.36)		1.16 (1.10 to 1.22)	

**Survey year**						
2010	30 498	7211 (23.6)	Ref		Ref	
2013	32 608	7775 (23.8)	1.01 (0.97 to 1.05)	0.0035	1.02 (0.98 to 1.06)	0.0409
2014	32 476	7401 (22.8)	0.95 (0.92 to 0.99)		0.97 (0.93 to 1.01)	

**Total**	95 582	22 387 (23.4)				

a The table is sorted in descending order of adjusted odds ratios. Rarer cancers without prior published evidence are denoted in bold italics.

b The Asian group excludes Chinese, which was treated as a separate category in all three surveys.

c *As classified by the* Index of Multiple Deprivation. *OR* = *odds ratio.*

There was also substantial variation between patients with any of the 12 rarer cancers of prime interest to this study (*P* <0.001 for test of variation between these 12 cancers). Specifically, patients with seven such cancers (small intestine, bone sarcoma, liver, gallbladder, cancer of unknown primary, soft-tissue sarcoma, and ureteric cancer) had proportions of ≥3 consultations that were >30.0%; whereas patients with five other rarer cancers (oropharyngeal, anal, parotid, penile, and oral cancer) had respective proportions of between 15% and 30% (Table 2). Multivariable analysis indicated concordant patterns (Table 2, Figure 2).

Relatedly, although bone and soft-tissue sarcomas tend to present with fairly specific symptoms (for example, bony or a soft-tissue lump), they principally affect teenagers and young adults, and are associated with relatively high crude proportions of multiple consultations.^20^ In multivariable analysis, however, after adjustment for age group and other patient characteristics, the odds of ≥3 consultations for these two cancers are notably reduced, although remaining comparatively high (Table 1).

**Figure 3.**
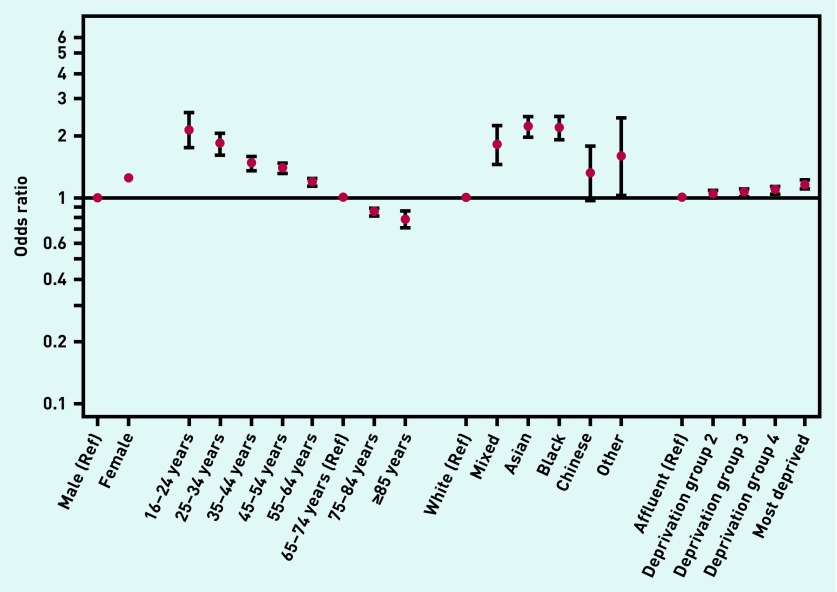
***Odds ratios for ≥3 GP consultations before hospital referral, by patient characteristic. Ref = reference***

Regarding sociodemographic variation, there was evidence of increasing frequency of ≥3 consultations with increasing deprivation (*P*<0.001): 26.5% versus 21.8% for patients in the groups of most and least deprivation respectively. As previously described,^11^ there was strong evidence for crude variation in pre-referral consultations by sex, age, and ethnicity (*P*<0.001 for all): with males, younger patients, and those from a minority ethnic group having a higher proportion of multiple pre-referral consultations (Table 2). Multivariable logistic regression analysis indicated concordant patterns of variation by patient characteristic, with the exception of a reversal of the sex difference (Table 2, Figure 3).

**Figure 4.**
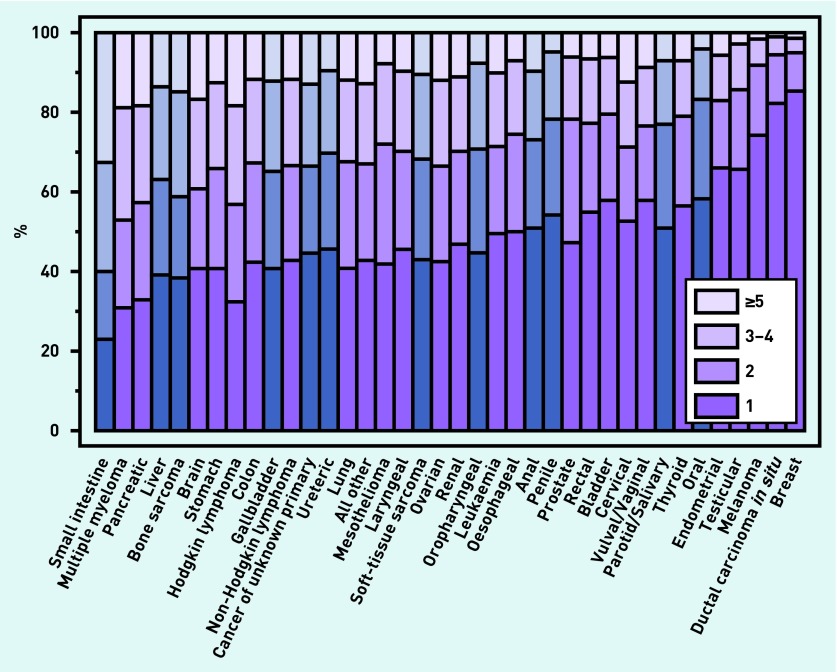
***Distribution of crude proportions of all categories of number of pre-referral consultation, by cancer diagnosis. The blue bars represent rarer cancers without prior relevant evidence.***

The distribution of the crude proportions of all categories of the number of pre-referral consultation by cancer diagnosis is shown in Figure 4 and Table 3. The overall pattern of variation by cancer diagnosis in respect of the binary measure of ≥3 consultations is, for the most part, similar to the pattern that would have been observed if alternative cut-off points (for example, ≥2 consultations or ≥5 consultations) had been used.

**Table 3.** Patients by cancer diagnosis and number of pre-diagnostic GP consultations^a^

	***n***	**Consultations before referral *n* (%)**			

		**1**	**2**	**3–4**	**≥5**
***Small intestine***	*218*	*50 (22.9)*	*37 (17.0)*	*60 (27.5)*	*71 (32.6)*
Multiple myeloma	2052	632 (30.8)	451 (22.0)	584 (28.5)	385 (18.8)
Pancreatic	1137	374 (32.9)	278 (24.5)	277 (24.4)	208 (18.3)
***Liver***	*451*	*177 (39.2)*	*107 (23.7)*	*105 (23.3)*	*62 (13.7)*
***Bone sarcoma***	*196*	*75 (38.3)*	*40 (20.4)*	*52 (26.5)*	*29 (14.8)*
Brain	474	193 (40.7)	95 (20.0)	107 (22.6)	79 (16.7)
Stomach	1828	746 (40.8)	459 (25.1)	394 (21.6)	229 (12.5)
Hodgkin lymphoma	969	314 (32.4)	238 (24.6)	239 (24.7)	178 (18.4)
Colon	8053	3424 (42.5)	1989 (24.7)	1713 (21.3)	927 (11.5)
***Gallbladder***	*66*	*27 (40.9)*	*16 (24.2)*	*15 (22.7)*	*8 (12.1)*
Non-Hodgkin lymphoma	5523	2372 (42.9)	1307 (23.7)	1187 (21.5)	657 (11.9)
***Cancer of unknown primary***	*3537*	*1585 (44.8)*	*763 (21.6)*	*733 (20.7)*	*456 (12.9)*
***Ureteric***	*331*	*151 (45.6)*	*80 (24.2)*	*69 (20.8)*	*31 (9.4)*
Lung	6405	2628 (41.0)	1709 (26.7)	1301 (20.3)	767 (12.0)
All other	2281	979 (42.9)	549 (24.1)	465 (20.4)	288 (12.6)
Mesothelioma	641	268 (41.8)	193 (30.1)	130 (20.3)	50 (7.8)
Laryngeal	667	303 (45.4)	166 (24.9)	133 (19.9)	65 (9.7)
***Soft-tissue sarcoma***	*508*	*218 (42.9)*	*128 (25.2)*	*109 (21.5)*	*53 (10.4)*
Ovarian	2717	1157 (42.6)	647 (23.8)	588 (21.6)	325 (12.0)
Renal	1436	673 (46.9)	332 (23.1)	273 (19.0)	158 (11.0)
***Oropharyngeal***	*1155*	*517 (44.8)*	*300 (26.0)*	*250 (21.6)*	*88 (7.6)*
Leukaemia	2043	1016 (49.7)	445 (21.8)	373 (18.3)	209 (10.2)
Oesophageal	2887	1437 (49.8)	713 (24.7)	539 (18.7)	198 (6.9)
***Anal***	*495*	*252 (50.9)*	*110 (22.2)*	*85 (17.2)*	*48 (9.7)*
***Penile***	*174*	*94 (54.0)*	*42 (24.1)*	*29 (16.7)*	*9 (5.2)*
Prostate	8538	4045 (47.4)	2630 (30.8)	1329 (15.6)	534 (6.3)
Rectum	5616	3094 (55.1)	1257 (22.4)	888 (15.8)	377 (6.7)
Bladder	6837	3961 (57.9)	1479 (21.6)	974 (14.2)	423 (6.2)
Cervical	706	372 (52.7)	131 (18.6)	115 (16.3)	88 (12.5)
Vulval/vaginal	405	235 (58.0)	75 (18.5)	59 (14.6)	36 (8.9)
***Parotid***	*140*	*71 (50.7)*	*37 (26.4)*	*22 (15.7)*	*10 (7.1)*
Thyroid	940	532 (56.6)	211 (22.4)	133 (14.1)	64 (6.8)
***Oral***	*567*	*330 (58.2)*	*143 (25.2)*	*71 (12.5)*	*23 (4.1)*
Endometrial	3255	2145 (65.9)	561 (17.2)	365 (11.2)	184 (5.7)
Testicular	665	437 (65.7)	132 (19.8)	77 (11.6)	19 (2.9)
Melanoma	3470	2576 (74.2)	605 (17.4)	233 (6.7)	56 (1.6)
Ductal carcinoma *in situ*	650	535 (82.3)	79 (12.2)	32 (4.9)	4 (0.6)
Breast	17 549	14 988 (85.4)	1678 (9.6)	639 (3.6)	244 (1.4)

a Cancer diagnoses are ordered as they appear in Table 2; rarer cancers without prior published evidence are denoted in bold italics.

## DISCUSSION

### Summary

The proportion of pre-referral consultations in patients subsequently diagnosed with several rarer cancers was particularly high (>30.0%) in patients with small intestine, bone sarcoma, liver, gallbladder, cancer of unknown primary, soft-tissue sarcoma, and ureteric cancer. The proportion of patients with any one of five rarer cancers (oropharyngeal, anal, parotid, penile, and oral cancer) who had ≥3 consultations was between 15% and 30%. Using a previously suggested classification, therefore, all of these cancers could be classified as either being harder to suspect or belonging to the intermediate diagnostic difficulty category.^10^

### Strengths and limitations

The main strengths of this study are its large sample size — enabling the examination of data on several rarer cancers with adequate precision — and the availability of data on patient characteristics, which allowed for the estimation of independent predictors of variation.

However, there are also potential limitations. Some patients may have recalled the number of relevant pre-referral consultations inaccurately, although prior evidence indicates that both patients and clinicians provide concordant frequencies of pre-referral consultations in patients with a given cancer.^8^ Further, the number of consultations that patients themselves judge relevant to cancer has high face validity, which cannot be dismissed. Relatedly, there was no information on actual circumstances surrounding the multiple consultation events, which may have included patient preferences for delayed referral or investigation, primary care-led investigations, and clinically justified expectant (‘safety-netting’) management, particularly for patients with non-specific symptoms.^10^,^17^ It is foolhardy to consider that multiple consultations represent suboptimal management: care is likely to have been concordant with good clinical practice in most circumstances, and multiple consultations represent scientific limitations in current medical knowledge, along with a lack of available, easy-to-use, reliable tests.^10^

Data relate to patients with recent hospital treatment for cancer (typically 3–6 months before survey participation). This sampling method, combined with non-response and early mortality patterns, results in a sample that is different in its cancer diagnosis and sociodemographic case mix compared with all incident patients with cancer in the population.^21^ As such, the reported proportions of ≥3 pre-referral consultations are not fully representative of incident patients with cancer and it is, therefore, recommended that interpretation focuses on patterns of relative variation between patients with different cancers and characteristics, rather than on the exact frequency of ≥3 pre-referral consultations by variable category.

### Comparison with existing literature

The authors know of no previous studies that examine the number of pre-referral consultations among patients with the studied rarer cancers of prior interest. However, in respect of patients with bone or soft-tissue sarcoma, the findings amplify those from a recent study of diagnostic pathways in patients with those cancers, indicating that most such patients who were diagnosed after a GP referral were referred to specialists non-urgently and without cancer being suspected as a diagnosis.^22^ Similarly, in respect of patients with cancer of unknown primary, the findings complement those of a recent Australian study that indicated delayed recognition of the importance of presenting symptoms in patients with those cancers.^23^

Previous evidence indicates that repeat consultations occur for between one-fifth and one-quarter of all patients with cancer, although this overall proportion varies greatly between patients with different cancers and characteristics.^8^,^11^ The present study extends prior relevant evidence on the frequency of multiple pre-diagnostic consultations to patients with 12 rarer cancers. Adding to previous evidence that indicates a higher risk of multiple pre-referral consultations in younger patients, those of minority ethnic groups, and males, the study presented here also identifies significantly higher risks of multiple consultations in patients of greater deprivation: prior relevant evidence was inconclusive due to power limitations.^11^

The frequency of multiple pre-diagnostic consultations varies greatly by cancer, being greatest for cancers that often present with symptoms that are common among patients consulting in primary care; consequently, these have low predictive value for cancer.^10^ For example, ≥3 pre-referral consultations occur in between one-third and one-half of all patients who are subsequently diagnosed with multiple myeloma or pancreatic cancer,^8^,^11^ which commonly present with musculoskeletal and abdominal pain, respectively, both of which are very common reasons for primary care consultations.^12^,^13^

This prior evidence provides insights into likely reasons for variation in the frequency of ≥3 pre-referral consultations in patients with rarer cancers: although some patients with small intestine, liver, and gallbladder cancers will present with ‘red-flag’ symptoms that have a relatively high predictive value, many will have symptoms such as diarrhoea or abdominal pain, which have relatively low predictive values for these (or other) cancers.^24^ Similarly, cancer of unknown primary typically presents with non-specific symptoms. Conversely, patients with oral and parotid cancer typically present with visible and/or palpable lesions (oral ulceration or facial lump) and, relatedly, have the lowest proportions of multiple consultations among the 12 rarer cancers described in this article. Therefore, concordant with prior evidence, the findings suggest that the frequency of multiple pre-referral consultations for different cancers reflects their ‘symptom signature’ (that is, the relative frequency and predictive value of the most common presenting symptoms); this observation holds true for both common and rarer cancers. Further, for a given cancer, the frequency of pre-referral consultations can be considered to denote its average diagnostic difficulty at first presentation to primary care.

Two further considerations can help to ascertain why the rarer cancers studied tend to fall within average or high diagnostic difficulty categories:^10^

- as rarer cancers are, by definition, very infrequent, doctors are particularly unfamiliar with their symptomatic presentations; and
- specifically for certain rarer cancers, greater incidence in younger age groups may exacerbate diagnostic difficulties as the predictive value of symptoms is particularly low compared with the same symptom in adults.

### Implications for research and practice

The findings underpin the need for the development, evaluation, and implementation of effective interventions aimed at decreasing the average number of pre-referral consultations in patients with cancer. In recent years, such interventions encompassed clinical practice guidelines for suspected cancer in primary care, the development and introduction of decision support tools during the consultation, and widening access to specialist investigations, such as imaging and endoscopy.^24^–^27^ Until recently, most interventions aimed at decreasing diagnostic intervals have focused on patients with relatively common cancers, such as initiatives aiming to increase endoscopic or imaging investigations for patients with suspected colorectal, lung, or ovarian cancer.^28^

Indeed, increased emphasis is being paid on system-wide approaches for supporting the diagnostic process. For example, ‘one-stop shop’ multispecialist diagnostic services for patients with serious, but unexplained, symptoms are being implemented in Denmark and the UK. Further, revised clinical guidelines for the referral of patients with suspected cancer have recently been introduced in England, encompassing a wide range of presenting symptoms for 34 different cancers in adulthood, including all cancers included in our study.^24^ Both the introduction of one-stop shop, diagnostic, clinic models and the implementation of new clinical guidelines encompassing a wider range of symptomatic presentations are likely to help improve diagnostic timeliness for patients with rarer cancers. In the longer term, the development of biomarker-enabled, point-of-care diagnostic tests is likely to be the most effective intervention for reducing multiple pre-referral consultations, particularly for cancers with a high level of diagnostic difficulty; and, therefore, for several rarer cancers.^10^

In spite of such developments, breakthroughs in diagnostic technologies are unlikely in the short term, and evidence on interventions such as decision support tools and new diagnostic care pathways is still in emergence. Safety netting patients with non-specific symptoms can have a role in improving diagnosis in the interim.^29^ The 2015 guidelines from the National Institute for Health and Care Excellence for the referral of patients with suspected cancer recommend the review for people with symptoms associated with increased risk of cancer who do not meet the criteria for referral or investigative action. The guidelines also encourage patient-initiated follow-up visits or arranging planned followup consultations in the presence of recurring, persistent, or worsening symptoms.^24^

These recommendations build on previous guidance from the Royal College of General Practitioners on safety netting.^30^ It should be noted that safety-netting approaches, by their nature, are likely to increase the number of consultations before referral or investigative actions in some patients; however, they can also shorten between-consultation intervals and improve the patient experience of the diagnostic process. Robust evaluation of safety-netting is nonetheless required, particularly as there is currently a plethora of definitions and variable practice. If effective, safety-netting approaches could be of particular benefit for patients subsequently diagnosed with rarer cancers.

This study has described the burden of pre-diagnostic consultations in primary care for patients with a range of rarer cancers. As implied by the respective proportions of patients having ≥3 consultations, most such cancers appear to have at least average, and often higher-than-average, diagnostic difficulty. The findings presented here could guide research and policy initiatives to increase diagnostic timeliness for patients with rarer cancers, including the introduction of decision support tools that encompass common relevant symptomatic presentations, the development of easily accessible multidisciplinary diagnostic services, and the wider introduction and evaluation of safety-netting approaches.
